# Evaluation of Machine Learning Model Performance in Diabetic Foot Ulcer: Retrospective Cohort Study

**DOI:** 10.2196/71994

**Published:** 2025-10-10

**Authors:** Veerle Y van Velze, Hendrico L Burger, Tim J van der Steenhoven, Hani Al-Ers, Lauren N Goncalves, Daniël Eefting, Willem-Jan J de Jong, Harm J Smeets, Janna C Specken Welleweerd, Joost R van der Vorst, Sandy Uchtmann, Robert Rissmann, Jaap F Hamming, Lampros Stergioulas, Koen EA van der Bogt

**Affiliations:** 1 Department of Surgery Haaglanden Medical Center The Hague The Netherlands; 2 Centre for Human Drug Research Leiden The Netherlands; 3 The Hague University of Applied Sciences The Hague, Zoetermeer The Netherlands; 4 University Vascular Centre West Leiden, The Hague, Delft The Netherlands; 5 Department of Vascular Surgery Leiden University Medical Center Leiden The Netherlands; 6 Department of Dermatology Leiden University Medical Center Leiden The Netherlands; 7 Leiden Academic Center for Drug Research Leiden The Netherlands

**Keywords:** machine learning, diabetic foot ulcer, complete wound healing, logistic regression, k-nearest neighbor, support vector machine, random forest, extreme gradient boosting, Bayesian additive regression trees, artificial neural network

## Abstract

**Background:**

Machine learning (ML) has shown great potential in recognizing complex disease patterns and supporting clinical decision-making. Diabetic foot ulcers (DFUs) represent a significant multifactorial medical problem with high incidence and severe outcomes, providing an ideal example for a comprehensive framework that encompasses all essential steps for implementing ML in a clinically relevant fashion.

**Objective:**

This paper aims to provide a framework for the proper use of ML algorithms to predict clinical outcomes of multifactorial diseases and their treatments.

**Methods:**

The comparison of ML models was performed on a DFU dataset. The selection of patient characteristics associated with wound healing was based on outcomes of statistical tests, that is, ANOVA and chi-square test, and validated on expert recommendations. Imputation and balancing of patient records were performed with MIDAS (Multiple Imputation with Denoising Autoencoders) Touch and adaptive synthetic sampling, respectively. Logistic regression, support vector machine (SVM), k-nearest neighbors, random forest (RF), extreme gradient boosting (XGBoost), Bayesian additive regression trees, and artificial neural network were trained, cross-validated, and optimized using random sampling on the patient dataset. To evaluate model calibration and clinical utility, calibration curves, Brier scores, and decision curve analysis (DCA) were performed.

**Results:**

The exploratory dataset consisted of 700 patient records with 199 variables. After dataset cleaning, the variables used for model training included age, smoking status, toe systolic pressure, blood pressure, oxygen saturation, hemoglobin, hemoglobin A_1c_, estimated glomerular filtration rate, wound location, diabetes type, Texas wound classification, neuropathy, and wound area measurement. The SVM obtained a stable accuracy of 0.853 (95% CI 0.810-0.896) with an area under the receiver operating characteristic curve of 0.922 (95% CI 0.889-0.955). The RF and XGBoost acquired an accuracy of 0.838 (95% CI 0.793-0.883) and 0.815 (95% CI 0.768-0.862), respectively, with areas under the receiver operating characteristic curve of 0.917 (95% CI 0.883-0.951) for RF and 0.889 (95% CI 0.849-0.929) for XGBoost. SVM, RF, and XGBoost were well-calibrated, with average Brier scores around 0.127 (SD 0.013). DCA showed that the SVM provided the highest net clinical benefit across relevant risk thresholds.

**Conclusions:**

Handling missing values, feature selection, and addressing class imbalance are critical components of the key steps in developing ML applications for clinical research. Seven models were selected for comparing their predictive power regarding complete wound healing, and each model representing a different branch in ML. In this initial DFU dataset used as an example, the SVM achieved the best performance in predicting clinical outcomes, followed by RF and XGBoost. The model’s calibration and clinical utility were determined through calibration curves, Brier scores, and DCA, demonstrating its potential relevance in clinical decision-making.

## Introduction

Machine learning (ML) has demonstrated remarkable efficacy in recognizing complex disease patterns and informing clinical decision-making, where conventional methods do not provide sufficient insights [1-4]. ML algorithms have been applied in various medical conditions, including cardiovascular diseases and diabetic foot ulcer (DFU) research. Previous papers have either provided an overview of potential ML models through a review or applied ML in data analyses [5]. However, there seems to be no prior medical study that has created a clear and detailed overview including examples of all the key steps generally involved in applying ML to datasets: data preprocessing, model selection, hyperparameter optimization, evaluation and comparison of model performance, and interpretation of results. Certain critical aspects of data analysis, such as addressing missing values and class imbalance, are frequently left out of the literature [6-11]. While a wide range of models can be used and compared, heavily imbalanced classes can still result in low area under the receiver operating characteristic curve (AUC) scores and accuracy [12]. Furthermore, previous studies compare at most 6 ML models, and no paper was found that explores the full spectrum of common ML models [6].

DFUs represent a complex and significant multifactorial medical problem, making these highly suitable for the application of ML. According to estimates from the International Diabetes Federation, in 2021, 537 million adults worldwide (10.5%) were living with diabetes mellitus, a number projected to escalate to 642 million by 2030. On average, 1 in 4 diabetics will experience a DFU in their lifetime, of which 22.3% (95% CI 15.3-29.2) eventually undergo a lower extremity amputation [13,14]. The personal and societal costs of DFUs and amputations are enormous, and as such, it is of utmost importance to correctly identify predictors of treatment response to optimize targeted interventions and prevent unnecessary treatments [15-18].

This study evaluates the use of 7 distinct ML models: logistic regression (LR), support vector machine (SVM), k-nearest neighbors (KNN), random forest (RF), extreme gradient boosting (XGBoost), Bayesian additive regression trees (BART), and artificial neural network (ANN). These models accommodate a wide range of regression, tree-based, probabilistic, and neural network methods, addressing higher complexity data. Moreover, LR, tree-based models, and BART provide a level of transparency that offers clear insights into the decision-making process.

Despite notable progress and research in ML, challenges persist in fully implementing all necessary steps. The multifactorial nature of DFU makes it a demanding and challenging case for evaluating the performance of ML models. By elucidating these methodologies, this paper aims to provide a framework for the proper use of ML algorithms to predict clinical outcomes of multifactorial diseases and their treatments, thereby assisting decision-making for clinicians.

## Methods

### Data Acquisition and Contextual Analysis

This study made use of a dataset containing records from 700 patients with a DFU due to neuropathy, ischemia, or both, who were treated at the Haaglanden Medical Center in The Hague, The Netherlands, between October 1, 2013, and October 1, 2021. Patients with a wound resulting from trauma, a surgical procedure, venous insufficiency, unguis incarnatus, or a wound located proximal to the malleoli were excluded. Structured into 71 unique categories, the dataset encapsulated 199 features per patient, reflecting a detailed record of each patient’s medical journey. These features spanned from generally available information to data acquired through medical examinations and posttreatment outcomes, all extracted from the electronic patient record, Healthcare Information Exchange. General demographic information was obtained from the cover page of the record, while medical history was retrieved from referral letters, outpatient reports, hospitalization records, and discharge summaries. Substance use was assessed through records from the departments of surgery, internal medicine, pulmonology, cardiology, and anesthesiology. Vital sign measurements and laboratory results were extracted from designated Healthcare Information Exchange sections, with averages calculated for the period in which the DFU was present. Medication use was determined based on prescription records, pharmacy verifications conducted during hospital admissions, and data from the National Exchange Point (ie, LSP). Information regarding the DFU was extracted from wound care center reports and hospitalization records.

A subset of the collected features was selected for analysis (see Feature Selection section). The patient’s age at the onset of the DFU was recorded in the database. Smoking encompassed the use of cigarettes, cigars, or other tobacco products. Wound location was categorized according to the angiosome concept [19]. Diabetes type was classified as type 1, noninsulin-dependent, and insulin-dependent diabetes. Neuropathy was defined as peripheral impairment of gnostic sensory function. Wound area was calculated by multiplying the average length by the average width of the DFU. Complete wound healing was defined as full closure of the DFU, with an intact epithelium and no signs of infection, necrosis, or fluid.

A key criterion for the inclusion of patient data in this retrospective cohort study was the completeness of the feature set. Patient data were included for analysis if it was entirely complete or if any missing values could be reliably imputed (see Handling Missing Values section). A significant proportion of patients did not undergo any form of treatment (ie, other than wound care or offloading), resulting in systematic presence of missing values in treatment-related features. To accommodate this and enhance the model’s predictive power, the selection of features for model training and application was intentionally restricted to those that are obtainable early in the patient’s care pathway (see Feature Selection section). In preparing the dataset for analysis, meticulous cleaning processes were used, which involved the exclusion of features based on redundancy in information, insufficiency in example frequency (eg, features related to rare treatments), and duplication of information through inverse representation.

### Handling Missing Values

Addressing missing values was paramount for the effective use of patient data in ML models. Imputation of missing values was used to mitigate substantial information loss (Table 1) [20,21]. Numerical variables necessitating imputation, such as average systolic and diastolic blood pressure, O_2_ saturation level, serum hemoglobin A_1c_ (HbA_1c_), hemoglobin (Hb), and estimated glomerular filtration rate (eGFR), were predominantly missing due to unmeasured instances rather than systemic bias. As such, to ensure statistical validity, a multiple imputation technique was applied to preserve the integrity of the data distribution. The technique used was matching imputation, specifically the MIDAS (Multiple Imputation with Denoising Autoencoders) Touch algorithm, due to its effectiveness in handling both numerical and categorical data gaps [22-24]. This algorithm identified data patterns to infer missing values through a linear regression model, with coefficients randomly selected from the feature distribution, and the process was iteratively refined to enhance imputation accuracy. Additionally, a weighted mean imputation strategy was incorporated that adopted 50 repetitions to ensure realistic and generalized imputation. Weight determination was based on the absolute difference between the median of the imputation and its vector value, followed by calculating the reciprocal of this difference. Weight values were capped at 2.0 to prevent excessive weighting.

**Table 1:** Features selected and transformed for model training and testing.

Feature name	Missing amount prior to imputation, n (%)	Data type	Transformation
Age	0 (0)	Numerical	*x* → √max(*x*)–*x*
Smoking	0 (0)	Categorical	N/A^a^
Systolic blood pressure	113 (16.1)	Numerical	N/A
Diastolic blood pressure	113 (16.1)	Numerical	N/A
Oxygen saturation level	154 (22)	Numerical	*x* → √max(*x*)–*x*
HbA_1c_^b^	82 (11.7)	Numerical	*x* → log (*x*)
Hemoglobin	0 (0)	Numerical	N/A
eGFR^c^	21 (3)	Numerical	*x* → √*x*
Wound location	0 (0)	Categorical	N/A
Toe systolic blood pressure	264 (37.7)	Numerical	N/A
Diabetes type	0 (0)	Categorical	N/A
Texas wound classification	0 (0)	Categorical	N/A
Neuropathy	0 (0)	Categorical	N/A
Wound area	80 (11.3)	Numerical	*x* → log (*x*)
Complete wound healing	0 (0)	Categorical	N/A

^a^N/A: not applicable.

^b^HbA_1c_: hemoglobin A_1c_.

^c^eGFR: estimated glomerular filtration rate.

### Feature Selection

In the preprocessing phase, feature selection played a crucial role in managing model complexity and ensuring adequate patient representation across different scenarios. This process relied on both statistical analysis and validation through literature to identify influential features, thus streamlining the model while preserving its predictive capability. Normalization of numerical values to approximate normal distributions was performed as a preliminary step to enhance statistical validity and facilitate effective feature selection. Normalization proved beneficial, where the normal distribution’s statistical properties were leveraged for analysis. Transformations were applied to nonnormally distributed variables, such as age, HbA_1c_, wound area, and eGFR, to align them with normal distribution characteristics. This helps the model recognize possible nonlinear correlations. To prevent imposing normality on the data, only common transformations such as lognormal, Box-Cox, and inverse transformations were applied. The selection of transformation depended on the form of skewness. Log and inverse transformations were applied to right-skewed data, while the Box-Cox transformation was used to address left-skewed data. Each transformation was based on visual inspection of the distribution and skewness prior to transformation. To check whether the distribution aids the transformation to normality, the Shapiro-Wilk test was performed after transformation with a threshold of 0.05. Feature selection was carried out using a 2-fold approach. First, relevant features were identified statistically. For each numeric feature, the group means were compared using ANOVA for normally distributed variables and Kruskal-Wallis for nonnormally distributed data. Chi-square tests were used for categorical variables. For both approaches, the 5% threshold was used to evaluate whether the feature should be included in the model. To prevent multicollinearity, only variables with a variance inflation factor below 5 were included. In the second time, expert recommendations (TJS, DE, WJJJ, JCSW, and KEAB) contributed to a second round of selection in the feature set, to include the role of clinical judgment in the final selection for context, and to resolve conflicting results, supported by literature on known predictors of DFU outcomes [25-32]. Age, smoking history, toe systolic pressure, blood pressure, oxygen saturation, Hb, HbA_1c_, eGFR, wound location, diabetes type, Texas wound classification, neuropathy, and wound area measurement were ultimately the features used to train the models.

### Addressing Class Imbalance

The balance of datasets is crucial for ML models, especially in medical diagnostics, where accurate identification of rare instances such as hemodialysis is significant despite their lower incidence in patient populations. Imbalanced datasets, with predominance of one class over another, can lead to bias in ML models by undervaluing the minority class, adversely affecting the model’s predictive performance on critical, less common outcomes [33,34]. A balanced dataset ensures equitable representation of all classes, mitigating bias and enhancing the model’s ability to capture the nuances of each category. To address class imbalance, sampling methods were used to adjust the class distribution within the dataset. The adaptive synthetic (ADASYN) oversampling method, a weighted variant of a synthetic minority oversampling technique, was used to balance the dataset between patients exhibiting full healing and those not, thereby correcting the initial skewness [35]. ADASYN focuses on oversampling in regions where the minority class is sparse, essentially making the algorithm focus on harder cases to predict. Furthermore, it improves on usual oversampling methods, as it uses a combination of KNN and linear interpolation, which helps preserve the statistical properties of the dataset. It was shown that ADASYN can improve the accuracy of a classifier in clinical trials [36]. Oversampling is a common practice in handling imbalanced classification problems. In numerous cases, it helps improve the accuracy of the model. However, in some studies, oversampling has shown no significant improvement in the performance of the classifier [37]. In oversampling, it is crucial that the original data are accurate, as any existing bias can be amplified through the oversampling process. Moreover, the smaller the number of minority class examples, the greater the risk of overfitting to synthetic patterns. Oversampling may also lead to the generation of illogical cases, which is why it is important to carefully inspect the synthetic data for such issues. To ensure the validity of synthetic patients with a nonhealing ulcer, logical tests were applied to detect any illogical oversampling instances. For example, when synthetic patient data fell outside of their respective prior distribution, this patient was deemed illogical and was then resampled. Validations included the ratio of systolic to diastolic blood pressure, the limitation to a single wound location, and the correct use of the Texas wound classification.

### Model Selection

This study evaluated 7 widely adopted, distinct ML models, LR, KNN, SVM, RF, XGBoost, BART, and ANN, for their efficacy in predicting DFU healing outcomes within 1 year. With these 7 models, an attempt was made to apply ML across the broadest range of possibilities. Specifically, the following range represents all ML models for the prediction of a (binary) classification.

LR, the baseline model, is widely recognized for its simplicity and effectiveness in medical binary classification tasks, although it may struggle with capturing nonlinear data relationships. LR is most appropriate when the relationship between features and the outcome is linear and when model interpretability and simplicity are prioritized. It performs effectively with smaller datasets and under conditions of minimal multicollinearity.

SVM excels in handling high-dimensional spaces through its capability to perform both linear and nonlinear classification; yet, it faces challenges with large datasets. Its capacity to handle nonlinear relationships via kernel functions makes it a robust choice for small- to medium-sized datasets where interpretability is less critical than accuracy.

KNN introduces flexibility by predicting outcomes based on the majority outcome of the most similar patients, adapting well to complex datasets at the cost of sensitivity to the number of patients and the distance metric. The KNN algorithm is well-suited for applications where no strong assumptions about the data distribution can be made. It is particularly effective for nonlinear decision boundaries and is most efficient on small- to medium-sized datasets due to its computational overhead during inference.

RF, an ensemble method, is praised for its ability to manage nonlinear data and variable interactions, offering insights into feature importance despite its comparatively lower interpretability. RF is a preferred method when dealing with datasets containing a large number of features or where overfitting is a concern. It effectively reduces variance and is versatile for both classification and regression tasks while also handling missing values well.

XGBoost stands out for its speed, performance, and robustness against overfitting through gradient boosted decision trees, although it demands meticulous hyperparameter tuning. XGBoost is highly effective for large-scale datasets with complex interactions and structured noise, particularly when computational efficiency and predictive performance are paramount. Its built-in handling of missing data and regularization makes it especially suitable for imbalanced datasets and high-stakes prediction tasks.

BART leverages probabilistic relationships to model dependencies between variables, making them useful for interpretative insights in complex datasets. Although BART can handle uncertainty effectively, it may require extensive domain knowledge and computational resources for accurate structure learning in large datasets. BART is recommended for problems requiring flexible nonparametric modeling with integrated uncertainty quantification and automatic variable selection.

ANN is powerful for capturing complex, nonlinear patterns in high-dimensional data, often outperforming other models in prediction accuracy for intricate tasks. However, their “black-box” nature limits transparency, making it difficult to interpret specific decision pathways, and they typically require substantial, highly varied data and computational power for optimal performance.

### Hyperparameter Optimization

The configuration of the hyperparameters affects the performance of the model, as the structure of the model and its capacity to generalize from the training data are significantly altered. These hyperparameters are configured independently of the data on which the model is trained, but the inherent meaning of the data does affect the predictive power of the configuration. Therefore, identifying the optimal configuration is crucial. A grid search was used to identify the best hyperparameter combination based on model accuracy. The grid search method iterated 1000 times over the model training and testing, recording the results of each iteration along with its hyperparameter setup and the prediction accuracy. In the event of missing values, class imbalance, or lack of data, it is essential to measure all performance metrics (see Evaluation and Comparison of Model Performance section). Only accuracy was ultimately used, as other metrics did not yield additional insight, and the other factors have been addressed. In cases where the hyperparameter range is large or computational time is a concern, heuristic search methods offer a more efficient alternative. For grid search specifically, each possibility of the configuration is included at least once. Visual confirmation was used to determine whether convergence toward the best hyperparameter combination was incidental or deterministic.

For LR, only the model weights were adjusted to reflect feature importance. For KNN, the k-number of neighbors was optimized to balance flexibility and overfitting. In SVM, cost and γ controlled the margin and kernel function, while degree and kernel type shaped the function itself.

In tree-based models like RF and XGBoost, hyperparameters such as the number of trees, maximum tree depth, and features per split regulated complexity and diversity of the ensemble trees. XGBoost further required learning rate η for convergence, split thresholds γ to prune weak splits, and regularization terms λ and α to reduce overfitting. BART retained default prior distributions for simplicity, although they can be adjusted when data-specific insights deem them unfit. For ANN, the number of hidden layers and neurons per layer was prioritized for their impact on model capacity and complexity.

### Evaluation and Comparison of Model Performance

As random variation in dataset partitioning can influence model outcomes, steps were taken to ensure that the results reflected both robustness and representativeness rather than chance. To address this challenge, repeated random split cross-validation was used by iteratively splitting the dataset and training the hyperparameter-optimized model 1000 times, ensuring that each possible configuration was included at least once in the grid search. In each iteration, the dataset was randomly divided into a unique training (70%) and testing (30%) set, which were kept separate to prevent data leakage. This iterative process allowed the model to be evaluated on different combinations of training and testing data, enhancing the reliability of its predictions. For each split, the model, configured with optimal hyperparameters, was then trained on the training set and evaluated on the testing set, both using patient characteristics. Following the prediction, the results were compared to the true values and fitted into a confusion matrix, with performance further quantified by average accuracy, specificity, precision, recall, and AUC. Finally, the iterative process provided not only a look into the average performance but also the variance. Low variance indicates the model’s ability to generalize well for patients outside of the training and testing data. Noteworthy indicators of model performance included accuracy for average performance, specificity for predictive power on patients with a nonhealing ulcer, and the considered balance between specificity and recall.

For the BART model, uncertainty quantification was enhanced through a density plot, providing greater transparency in the decision-making process. The density plot visualizes the probability distribution of wound healing. The peak of the plot indicates where the model is most confident in its classification. A red line marks the threshold, the probability level used to distinguish between healing and nonhealing outcomes. Predicted probabilities exceeding this threshold are classified as healing, while those falling below it are classified as nonhealing.

A calibration curve was used to ensure that the predicted probabilities reflect true outcome likelihoods. This curve compares predicted probabilities to observed outcomes to detect systematic over- or underestimation. To quantitatively evaluate calibration, Brier scores were calculated. The Brier score, ranging from 0 to 1, reflects the model’s ability to estimate true outcome probabilities with high certainty, with lower values indicating better calibration.

Decision curve analysis (DCA) was used to evaluate the clinical use of the ML models (see Model Selection section) [38]. DCA measures the net benefit of a model dependent on risk threshold probability. It displays the most beneficial decision-making strategy corresponding to a patient risk probability. For instance, in a patient with a DFU who receives a predicted 85% risk of nonhealing, a clinical decision rule that recommends amputation for any risk exceeding 80% would lead to an amputation. If, in reality, the DFU would have healed without surgical intervention, this decision would constitute overtreatment. DCA allows such trade-offs to be visualized and quantified across a range of thresholds, thereby supporting the selection of a decision threshold that yields the highest overall clinical benefit.

### Dataset Size

A common challenge in ML training is the need for a sufficiently large dataset to effectively train a classification model. In practical applications, obtaining such data is difficult and time-consuming. Hence, the improvement in model performance relative to dataset size was measured. The selected ML models were trained with randomly sampled, evenly classified train and test datasets. The performance results were visualized together with a learning curve illustrating the dataset size expansion required to achieve the anticipated performance level. Learning curves are a practical tool for evaluating the relationship between model performance and dataset size. These curves help determine the extent to which additional training data might enhance model performance.

### Ethical Considerations

The study was conducted in accordance with the principles of the World Medical Association Declaration of Helsinki. The independent Medical Ethics Review Committee Southwest Holland reviewed the study and determined that it does not fall within the scope of the Medical Research Involving Human Subjects Act (approval number 18-038), as it does not constitute scientific research as defined in article 1, paragraph 1, subsection b of the Medical Research Involving Human Subjects Act. The study consists of a retrospective chart review rather than research in which individuals are subjected to interventions or imposed behavioral regulations. Additionally, the scientific and general board of directors of the Haaglanden Medical Center approved the execution of the study and granted an exemption from obtaining informed consent due to the retrospective nature of data collection and the exclusive use of anonymized data. This decision was based on the minimal risk to patients, the impracticality of contacting patients, and the strict safeguards implemented to protect privacy. The data were derived from electronic patient records and documented anonymously within the Castor medical web application. All data handling was conducted in compliance with the General Data Protection Regulation. Access to the dataset was restricted to authorized members of the research team, which was stored on a protected hospital server with controlled access. No identifiable information was used in the analyses or presented in the results. As the study relied exclusively on retrospective and anonymized data, no patients were contacted, and no financial or other compensation was provided.

## Results

The final dataset used for prediction and analysis contained information from 900 patients. After applying imputation and oversampling techniques for incomplete patient records, 308 (44%) patients were recovered for model training (Table 2). The original dataset comprised 700 patients, with 427 (61%) representing cases of complete wound healing. Through ADASYN, approximately 200 synthetic patient records with a nonhealing ulcer were generated, equalizing the representation of both classes to 450 (50%) patients.

**Table 2:** Statistical validity before and after imputation of empty values.

Feature	Before imputation, mean (SD)	After imputation, mean (SD)
Diastolic blood pressure (mm Hg)	69 (12)	70 (12)
O_2_ saturation (%)	97 (2)	97 (2)
Serum eGFR^a^ (mL/min/1.73 m^2^)	66 (33)	66 (33)
Serum hemoglobin (mmol/L)	7.8 (1.2)	7.8 (1.1)
Serum HbA_1c_^b^ (mmol/mol)	69 (19)	69 (18)
Systolic blood pressure (mm Hg)	137 (18)	137 (16)
Toe systolic blood pressure (mm Hg)	77 (46)	76 (39)
Wound area measurement (cm^2^)	4.1 (11.6)	3.8 (11.0)

^a^eGFR: estimated glomerular filtration rate.

^b^HbA_1c_: hemoglobin A_1c_.

**Figure 1:**
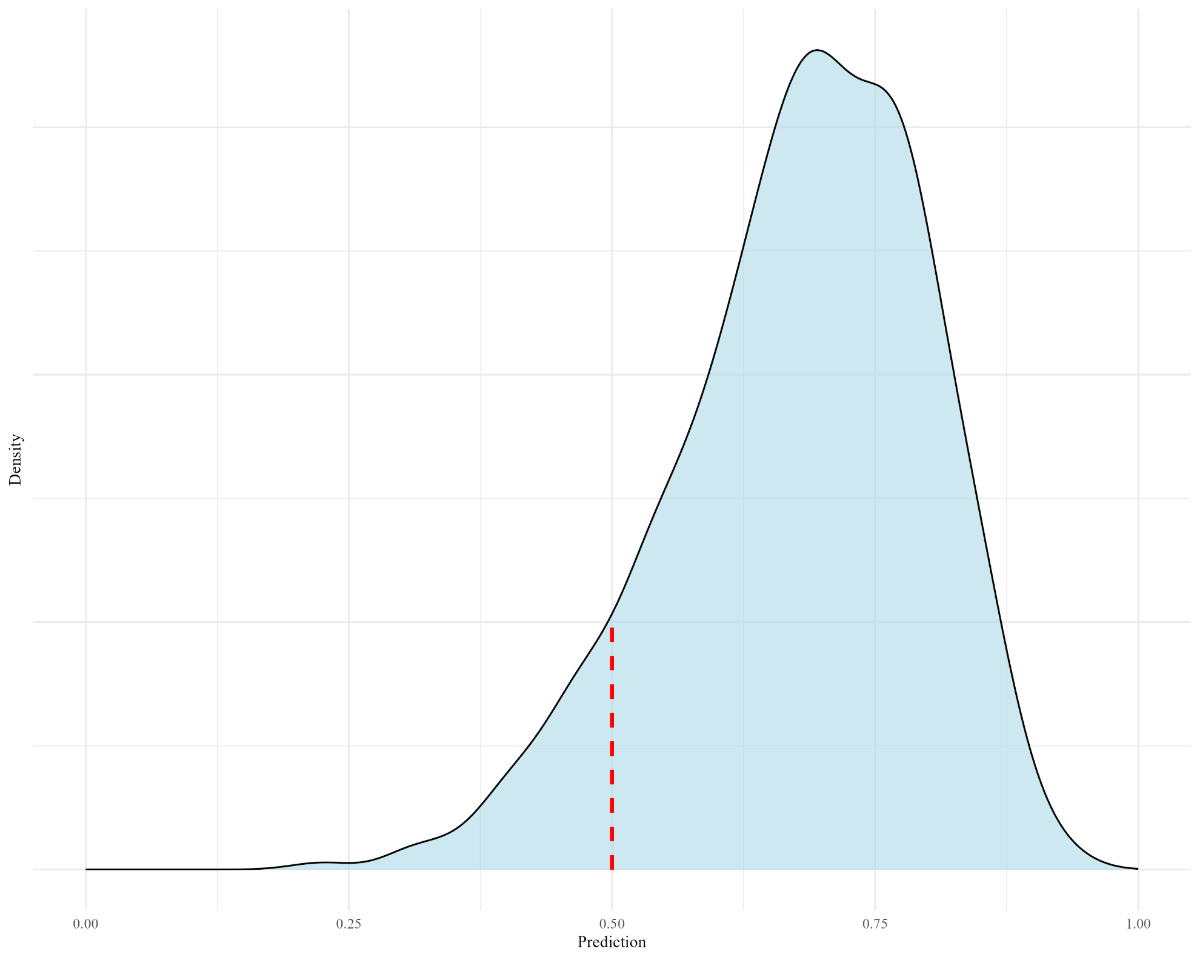
Density plot for uncertainty quantification of a Bayesian additive regression trees model prediction.

**Figure 2:**
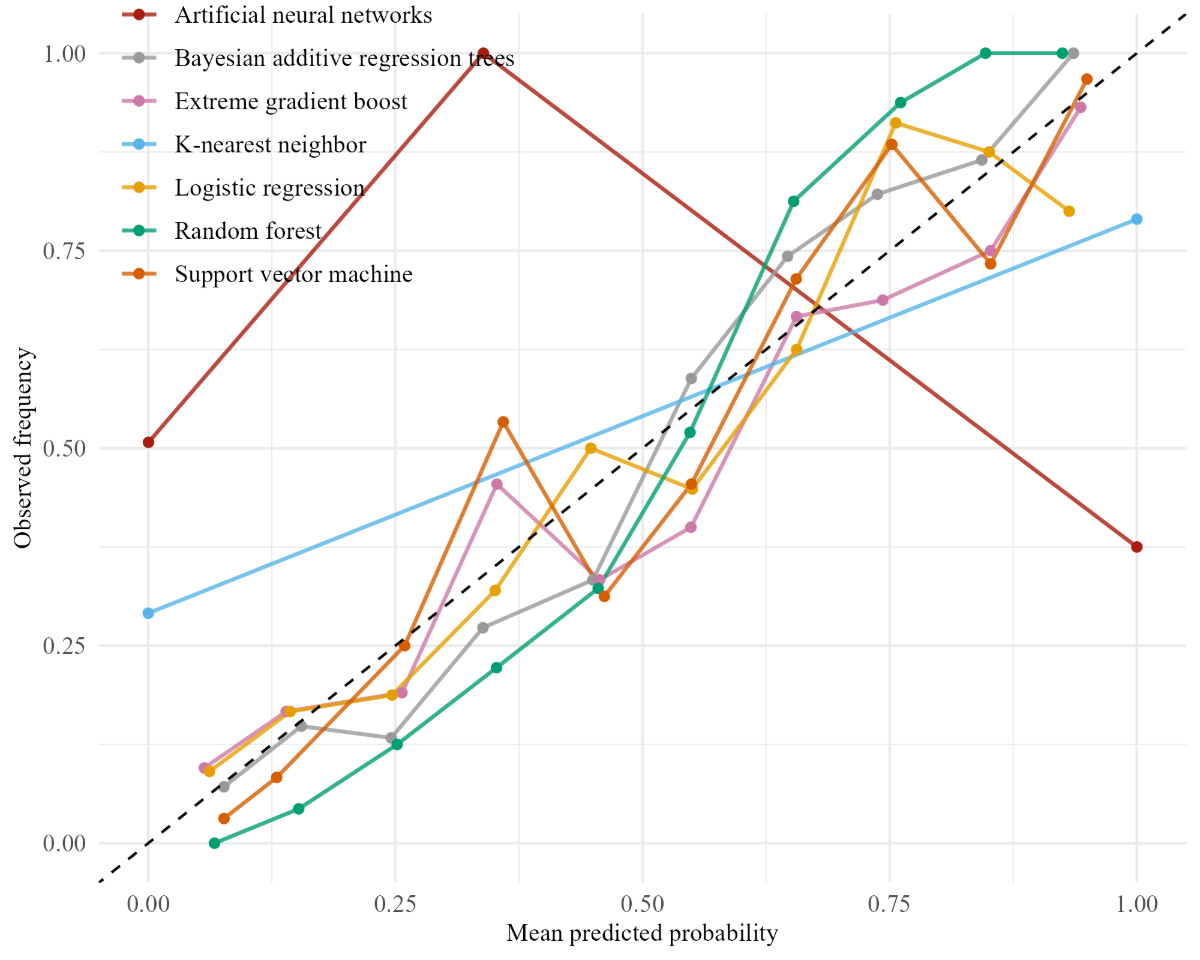
Calibration curves: calibration performance of the machine learning models compared to the ideal calibration represented by the diagonal dotted black line.

Feature selection of the dataset yielded the features that are linearly correlated with wound healing (α=.05) and used in the prediction of DFU healing (see Feature Selection section). Only wound location contained variance inflation factor values greater than 5 due to the wide variety of locations. Seven models, LR, KNN, SVM, RF, XGBoost, BART, and ANN, were optimized with a grid search set within the most commonly used boundaries (see Table 3 for hyperparameters) [39]. These models were trained and tested, resulting in the following outcomes. The validation of model training demonstrated a high predictive power with an average accuracy of 0.794 (SD 0.023). However, notable performance differences existed when comparing models against their respective baselines. Feature importance measured by both the RF and XGBoost models revealed critical predictors for DFU healing outcomes. These predictors were determined by their use in decision-making. Key predictors highlighted by both models included toe systolic pressure on the affected side, Hb, wound area, age, systolic blood pressure, and diastolic blood pressure. Furthermore, BART offered enhanced insights into uncertainty quantification through a density plot, contributing to greater transparency in the decision-making process. The density plot represents the probability of wound healing for a randomly selected patient, with a 69% likelihood that healing will occur (Figure 1). Table 4 shows the model performance averaged over 1000 iterations in terms of accuracy, specificity, precision, recall, and AUC metrics. SVM achieved the highest performance with an accuracy of 0.853, followed by RF (accuracy=0.838) and XGBoost (accuracy=0.815). KNN (accuracy=0.789) and ANN (accuracy=0.789) scored lower, followed by BART (accuracy=0.748), with LR (accuracy=0.725) scoring the least in overall performance. The reliability of the predicted probabilities was assessed using calibration curves (Figure 2). The ideal calibration is represented by the diagonal dotted black line, indicating perfect alignment between predicted and actual probabilities. Deviations from this line indicate miscalibration. Curves below the diagonal suggest overconfidence (ie, predicted probabilities are too high), while curves above the diagonal indicate underconfidence (ie, predicted probabilities are too low). The Brier scores indicate that SVM, RF, and XGBoost are better at class discrimination, whereas LR, KNN, and ANN exhibit less reliable probability estimates (Table 4).

**Table 3:** Results of the model hyperparameter tuning.

Model		Hyperparameters (lower bound-upper bound)
**Logistic regression**		
	—^a^	—
**Support vector machine**		
	Gamma	0.1 (0.1-10)
	Cost	7.1 (0.1-100)
	Degree	3 (1-5)
	Kernel	Radial basis
**K-nearest neighbor**		
	K	1 (1-10)
**Random forest**		
	Number of trees	230 (10-300)
	Depth	90 (10-100)
	Features	3 (1-25)
**Extreme gradient boosting**		
	Number of trees	30 (10-300)
	Depth	16 (10-100)
	Eta	0.23 (0.01-0.4)
	Gamma	0.19 (0.01-0.2)
	Lambda	1.60 (0.1-2)
	Alpha	0.30 (0.1-2)
**Bayesian additive regression trees**		
	Number of trees	30 (10-100)
	Depth	90 (10-100)
**Artificial neural network**		
	Layers	2 (1-5)
	Neurons	16. 8 (64.2-32.2)
	Threshold	0.001 (0.1-0.001)

^a^Not available.

**Table 4:** Model performance metrics with 95% CI.

Model	Accuracy (95% CI)	Specificity (95% CI)	Precision (95% CI)	Recall (95% CI)	AUC^a^ (95% CI)	Brier score (95% CI)
Logistic regression	0.725 (0.679-0.771)	0.711 (0.628-0.794)	0.723 (0.669-0.793)	0.739 (0.666-0.812)	0.794 (0.751-0.837)	0.179 (0.156-0.202)
Support vector machine	0.853 (0.810-0.896)	0.846 (0.772-0.920)	0.851 (0.792-0.910)	0.860 (0.799-0.921)	0.922 (0.889-0.955)	0.111 (0.087-0.135)
K-nearest neighbor	0.789 (0.744-0.834)	0.891 (0.831-0.951)	0.870 (0.808-0.932)	0.692 (0.620-0.764)	0.792 (0.747-0.837	0.131 (0.131-0.131)
Random forest	0.838 (0.793-0.883)	0.850 (0.781-0.919)	0.853 (0.793-0.910)	0.826 (0.761-0.891)	0.917 (0.883-0.951)	0.133 (0.115-0.151)
Extreme gradient boosting	0.815 (0.768-0.862)	0.822 (0.747-0.897)	0.827 (0.768-0.886)	0.809 (0.742-0.876)	0.889 (0.849-0.929)	0.138 (0.105-0.171)
Bayesian additive regression trees	0.748 (0.701-0.795)	0.736 (0.661-0.811)	0.746 (0.692-0.800)	0.762 (0.688-0.836)	0.749 (0.702-0.796)	0.169 (0.129-0.190)
Artificial neural network	0.789 (0.736-0.842)	0.770 (0.703-0.837)	0.761 (0.654-0.868)	0.811 (0.730-0.892)	0.793 (0.744-0.842)	0.202 (0.153-0.251)

^a^AUC: area under the receiver operating characteristic curve.

**Figure 3:**
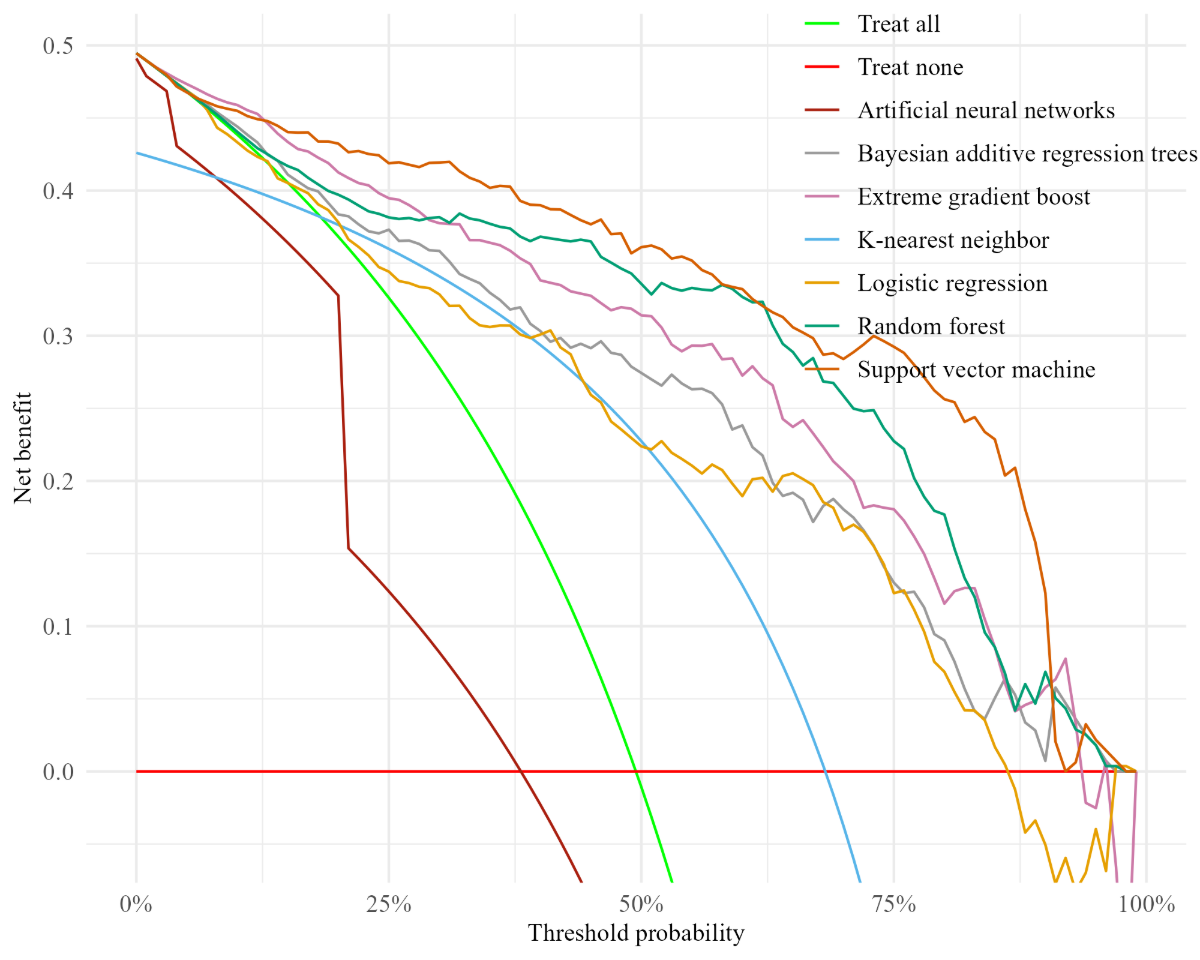
Decision curve analysis: correlation between net benefit and patient risk threshold probability of model predictions.

The DCA showed the benefit of each ML model in the application of complete wound healing within 1 year (Figure 3). To facilitate interpretation, the threshold probability was modeled to the estimated risk of patients not achieving complete wound healing within 1 year. Overall, the SVM consistently outperformed both the treat all and treat none strategies, as well as the other ML models evaluated. This advantage was particularly evident at the 75% risk threshold, where the SVM demonstrated the highest net benefit. With the exception of the ANN, the curves remained distant from the extremes of treating all or no patients, demonstrating clear clinical benefit. The ANN curve remained close to the treat all baseline, indicating limited net benefit compared to the other models. The analysis highlighted the potential of ML to support and enhance clinical decision-making in DFU care, especially between the higher risk threshold values (0.2-1.0).

**Figure 4:**
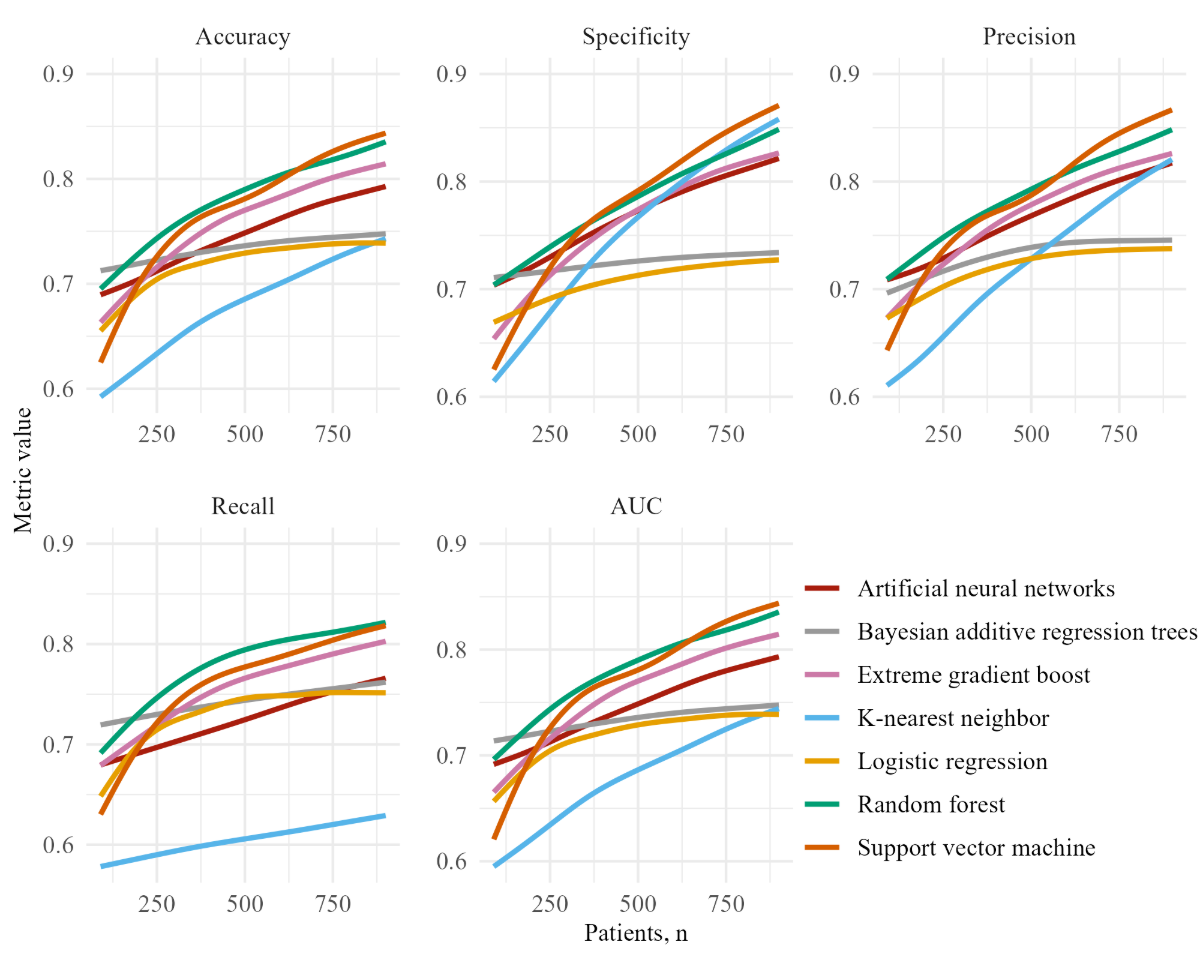
Learning curves: correlation between model performance metrics and dataset size. AUC: area under the receiver operating characteristic curve.

Finally, the model’s performance in relation to the dataset size provided valuable insights into potential improvements in average performance (Figure 4). While the baseline LR reached its peak accuracy at approximately 73%, the SVM, RF, XGBoost, and ANN exhibited promising signs of improvement with increasing dataset size.

## Discussion

### Principal Findings

This paper offered a structured framework for applying ML models in clinical research. Using ML in a dataset necessitated several fundamental steps: data acquisition, contextual analysis, handling missing values, feature selection, addressing class imbalance, model selection, hyperparameter optimization, evaluation and comparison of model performance, and interpretation of results. As ML models become more accurate with more examples in a database, developing a good model requires significant computational power. The choice of different statistical methods was related to the number of available variables, the size of the studied population, and the outcomes of interest. This study demonstrated that SVM, RF, and XGBoost achieved the best performance in predicting outcomes from a dataset related to a multifactorial disease.

Imputation with low variance in feature distribution ensured statistical coherence, which is essential in medical research. This coherence supports the accuracy of patient representation and validates the reliability of the results, providing a statistically coherent foundation for model training.

Preprocessing and feature selection resulted in a clean dataset with features that were linearly correlated with complete wound healing within a year. These features included age, smoking status, toe systolic pressure, blood pressure, oxygen saturation, Hb, HbA_1c_, eGFR, wound location, diabetes type, Texas wound classification, neuropathy, and wound area measurement. These features aligned with findings reported in prior literature [40,41]. Seven models were selected for comparing their predictive power regarding complete wound healing, and each model representing a different branch in ML [42,43]. Each model provided distinct characteristics and unique insights, suggesting that combining multiple models could be a valuable approach to enhance predictive accuracy and interpretability. This study’s comparative approach sought not only to discern the optimal models for predicting DFU healing but also endeavored to deepen the understanding of the underlying factors influencing these outcomes. Feature importance therefore played a pivotal role in understanding the relative significance of features within each algorithm. To achieve this, the influence of features on model predictions was explored. Specifically, the feature importance of the RF and XGBoost models was taken into account due to their ability to measure nonlinear correlations. Upon comparing overall predictive scores, SVM performed best. While the baseline LR model and BART plateaued at 0.75 accuracy, KNN, RF, XGBoost, ANN, and even SVM itself continued to show signs of improvement when trained on an increasing dataset size. Although BART did not achieve high accuracy, it demonstrated value by providing predictive certainty, which is essential for supporting informed decision-making, assessing risk, and enhancing transparency in complex scenarios, where achieving complete accuracy is not feasible. The Brier scores, which quantify overall model calibration quality, supported the results observed in model performance. SVM achieved the lowest Brier score, indicating that the predicted probabilities were also well-calibrated and were able to discriminate between classes. These findings are further reinforced by the DCA, which demonstrated that ML models, especially SVM, consistently yielded the highest net benefit across the full range of threshold probabilities, validating clinical usability. The limited performance of the ANN likely stems from its inclination to predict predominantly a single class, reflecting both insufficient training data and difficulty in capturing complex patterns within the dataset. To realize improved model performances for KNN, RF, XGBoost, ANN, and SVM, we aim to include more patients before moving toward clinical applicability.

### Imperative Steps in Clinical ML

The fundamental steps necessary for applying ML to a dataset are elaborated on in the Methods section. Each step serves a distinct purpose, and omitting any of them may lead to detrimental consequences. For instance, handling missing values is imperative, as ignoring them can result in an inaccurate representation of the patient population, distorting the model’s output and leading to incorrect predictions that fail to reflect the true clinical situation. Similarly, neglecting class imbalance can cause the model to perform poorly in predicting the rarer class, which may lead to incorrect treatment decisions in clinical practice. Additionally, choosing the wrong model or failing to optimize hyperparameters can prevent the model from generalizing well to new data, ultimately undermining its clinical applicability.

### Comparison with Prior Work

In other DFU-related studies, 1 or a combination of up to 6 different ML models were used to predict disease outcomes [6,11,25,44-49]. In addition to the 7 models selected for this paper, various papers proposed the use of specialized model variants, ensembles, or hybrids. Some examples are stacking C, bagging, Adaboost, and light gradient boosting machine [42,45,50]. We chose to exclude these models to uphold the simplicity and clarity of our application.

### Transferability Across Clinical Domains

The methodological framework outlined in this paper is intended to be broadly applicable across a wide range of clinical domains and is particularly well-suited for multifactorial diseases, such as cardiovascular diseases, oncology, autoimmune disorders, neurodegenerative diseases, and psychiatric conditions. These disorders are often characterized by complex, nonlinear interactions between numerous variables. ML models offer a distinct advantage over conventional analytical approaches by effectively identifying and prioritizing relevant features. Moreover, there is growing recognition that environmental and geographical factors significantly influence disease outcomes, contributing to an increasing number of relevant variables in datasets. To ensure optimal model performance and enhance clinical applicability, it is crucial to ensure that a database comprises a sufficiently large cohort with an adequate number of variables. Potential challenges in applying the framework to other clinical conditions include the presence of variables with many distinct classes or free-text fields, rare outcomes occurring in only a small subset of the population, a dataset with a large proportion of imaging data, and structurally missing or incomplete data. In certain domains, temporal dynamics play a critical role, which may necessitate the use of more advanced modeling techniques to appropriately capture and analyze time-dependent patterns.

### Strengths and Limitations

For this paper, a near-complete dataset was compiled, encompassing all consecutive patients from one of the largest hospitals in the Netherlands regarding DFU management, comprising 199 variables. Substantial effort was dedicated to collecting and curating the data to ensure its completeness and quality. Additionally, 7 different ML models were applied, making this paper a strong representation of the current capabilities of ML in this domain.

Leveraging this extensive dataset and the application of multiple ML models, this study highlights the advantages of ML over conventional statistical methods in DFU management. Besides their ability to process large amounts of data, these ML models offer several key benefits. First, the models can identify complex, nonlinear relationships between variables that may be overlooked by conventional methods. In complex datasets such as the one used in this paper, interactions between different factors can be subtle. Second, the ML models used provided alternative methods to extract relevant features from data, helping researchers identify important predictive factors that might have otherwise been overlooked. Certain variables may unexpectedly prove important, as manually identifying the most relevant features in high-dimensional datasets can be challenging. Unlike traditional approaches, which rely on human intuition and domain knowledge, both of which can introduce bias, ML models use data-driven techniques to determine feature importance objectively. Third, the models effectively handled missing data through imputation, enhancing data usability and improving predictive accuracy. It is important to note, however, that imputation should only be applied when a small proportion of data is missing. Applying imputation to a large number of missing values can significantly distort the dataset, introduce bias, and compromise the validity and reliability of the resulting predictive models. ML models can adapt and improve, as more data become available, continuously refining and optimizing predictions. Integrating ML models into clinical decision support systems can offer objective, data-driven insights for health care providers and patients, allowing informed personalized decisions, such as the option to wait longer for wound healing or to pursue a different path in DFU treatment such as amputation and early revalidation when muscle mass is not yet decreased because of prolonged immobilization.

The retrospective nature of this database presents a limitation for this study, as some data were not recorded for a portion of the patient cohort. In this regard, it would have been ideal to analyze a prospective dataset. Furthermore, clinical applicability would be more profound with a larger database. This could not have been anticipated prior to the analysis, as the learning curves demonstrate an increase in performance up to at least 900 patients (Figure 4). Learning curves provide valuable insights into how much the model’s performance might improve with additional training data, helping to determine whether collecting more data is beneficial or if the model has reached its performance plateau. When there is still room for improvement, one must be cautious of overfitting, where models overly adapt to training data, performing well on training data but poorly generalizing to new, unseen data. This can result in inaccurate predictions and decision-making in practice. This limitation may be overcome by providing sufficient training data, reducing the likelihood that unseen data will yield a different outcome. In addition, implementing cross-validation helps assess generalizable performance, while avoiding data leakage, ensuring balanced class distributions, and minimizing the number of features further contribute to reducing overfitting. Moreover, there was limited availability of specific variables, such as having a rare condition. These variables could not be included in the feature selection, as their absence can lead to biases in the models and inaccurate predictions. Therefore, clinical applicability needs to be validated in an extended database.

To ensure the added value of ML models in clinical practice, it is an important role of computer data scientists to formulate quality standards for clinical decision models. In addition, a careful collaboration between the computer scientists, statisticians, and clinicians is necessary to identify suitable outcomes in clinical, imaging, and biochemical parameters. For example, one would be most interested in factors that can be clinically modified or that strongly predict good or absolutely worst outcomes. Moreover, clinical interpretation of findings from ML models is clearly needed. For example, Hb levels showed a strong relationship with wound healing outcomes in this study. However, while poor wound healing could be a direct result of anemia, anemia may also be a consequence of a generally ill patient because of a chronic wound.

### Future Research

The next step will focus on obtaining more than 1000 complete patient records in the DFU dataset to provide clinically relevant features and develop a combined ML prediction model with an accuracy exceeding 90%.

### Conclusions

This study aimed to provide a framework for using ML models to investigate factors influencing a multifactorial disease. Taken together, the steps involved in the process include data acquisition, contextual analysis, handling missing values, feature selection, addressing class imbalance, model selection, hyperparameter optimization, evaluation and comparison of model performance, and interpretation of results. By using 7 different ML models, we have shown quality control for each method. In the pilot DFU dataset used as an example, the SVM performed the best, followed by RF and XGBoost. By incorporating our framework, explanations to model behavior can be structurally approached, providing better insights compared to metric values alone.

## Abbreviations

ADASYN: adaptive synthetic
ANN: artificial neural network
AUC: area under the receiver operating characteristic curve
BART: Bayesian additive regression trees
DCA: decision curve analysis
DFU: diabetic foot ulcer
eGFR: estimated glomerular filtration rate
Hb: hemoglobin
HbA1c: hemoglobin A1c
KNN: k-nearest neighbor
LR: logistic regression
MIDAS: Multiple Imputation with Denoising Autoencoders
ML: machine learning
RF: random forest
SVM: support vector machine
XGBoost: extreme gradient boosting

## Data Availability

The dataset generated and analyzed during this study is not publicly available due to the protection of patient privacy but are available from the corresponding author on reasonable request. All analyses were performed on the Windows 11 operating system using RStudio (version 4.4.1; Posit PBC) and open-source packages. The complete code and workflow are available on GitHub, ensuring full reproducibility [51].
